# Zika Virus Infection, Philippines, 2012

**DOI:** 10.3201/eid2609.190896

**Published:** 2020-09

**Authors:** Corazon Cerilla Buerano, Lady-Anne Suarez Pangilinan, Maria Terrese Alonzo Dimamay, Cynthia Abad Mapua, Mark Pierre Sijo Dimamay, Ronald Roll Matias, Filipinas Florendo Natividad, Maria Luisa de Guzman Daroy, Futoshi Hasebe, Kouichi Morita, Meng Ling Moi

**Affiliations:** St. Luke’s Medical Center, Quezon City, Philippines (C.C. Buerano, L.-A.S. Pangilinan, M.T.A. Dimamay, C.A. Mapua, M.P.S. Dimamay, R.R. Matias, F.F. Natividad);; St. Luke’s Medical Center College of Medicine, Quezon City (M.L.G. Daroy);; WHO Collaborating Centre for Reference and Research on Tropical and Emerging Viral Diseases, Institute of Tropical Medicine (NEKKEN), Nagasaki, Japan (F. Hasebe, K. Morita, M.L. Moi)

**Keywords:** Zika virus, viruses, febrile illness, vector-borne infections, mosquitoes, Philippines

**To the Editor:** Alera et al. described a 2012 case of Zika virus infection in the Philippines (*1*). In 2007, a Zika virus outbreak occurred in Yap, Micronesia, possibly caused by travelers from the Philippines (*2*). Zika virus infections were reported in the Philippines in 1953, 2012, and 2016 (*3*). Although frequent travel exchange between Yap and the Philippines could be a possible transmission route, no data on Zika virus infection were recorded in the Philippines between 1953 and 2012.

We detected Zika virus infection in 1 (0.75%) of 134 febrile, non–dengue infected patients at St. Luke’s Medical Center (Quezon City, the Philippines) during 2010–2015 by subjecting patient serum samples to serological and molecular tests. Ethics clearance (reference no. 19042) for this study was given by St. Luke’s Medical Center Institutional Ethics Review Committee. The only patient who tested positive for Zika virus was a 31-year-old woman diagnosed with an upper respiratory tract infection in 2010. Because of her work, she might not have traveled internationally. We obtained her serum sample on day 3 of fever. She did not have a rash or arthralgia. Although we did not isolate Zika virus according to guidelines (*4*), we confirmed infection using other techniques. The patient’s serum sample tested positive for Zika virus RNA, IgM against Zika virus, and neutralizing antibodies against Zika virus by using a plaque reduction neutralization test to neutralize 50% of plaques (PRNT_50_) (PRNT_50_ Zika virus = 1:80, PRNT_50_ dengue virus serotypes 1–4 <1:10). The sample tested negative for IgM against dengue and Japanese encephalitis viruses but positive for IgG against Zika virus nonstructural protein 1. These results suggest local Zika virus infection in the Philippines since at least 2010, 2 years earlier than the previously reported infection (*1*).
